# A Folding-Based Electrochemical Aptasensor for the Single-Step Detection of the SARS-CoV-2 Spike Protein

**DOI:** 10.1021/acsami.2c02405

**Published:** 2022-04-21

**Authors:** Federica Curti, Simone Fortunati, Wolfgang Knoll, Marco Giannetto, Roberto Corradini, Alessandro Bertucci, Maria Careri

**Affiliations:** † Department of Chemistry, Life Sciences and Environmental Sustainability, University of Parma, 43124 Parma, Italy; ‡ Biosensor Technologies, AIT-Austrian Institute of Technology GmbH, Konrad-Lorenz-Straße 24, 3430 Tulln an der Donau, Austria; § Department of Scientific Coordination and Management, Danube Private University, A-3500 Krems, Austria

**Keywords:** DNA aptamer, electrochemical sensors, DNA nanotechnology, COVID-19, single-walled carbon nanotubes

## Abstract

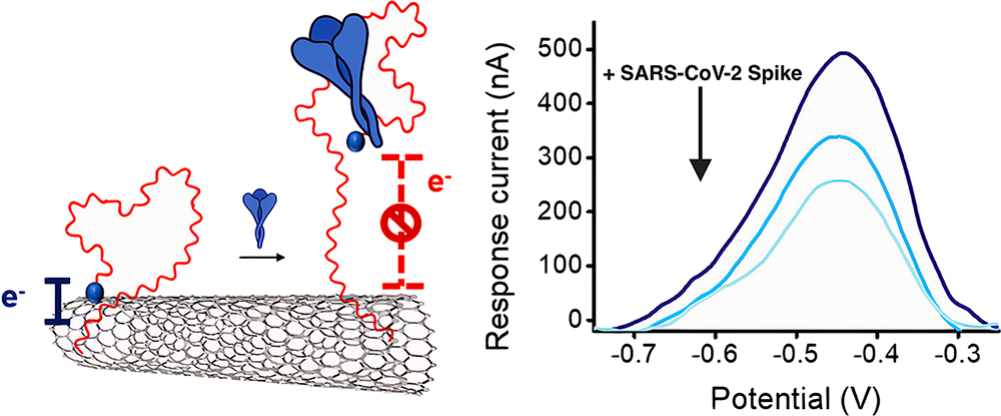

Efficient and timely testing has taken center stage in the management, control, and monitoring of the current COVID-19 pandemic. Simple, rapid, cost-effective diagnostics are needed that can complement current polymerase chain reaction-based methods and lateral flow immunoassays. Here, we report the development of an electrochemical sensing platform based on single-walled carbon nanotube screen-printed electrodes (SWCNT-SPEs) functionalized with a redox-tagged DNA aptamer that specifically binds to the receptor binding domain of the SARS-CoV-2 spike protein S1 subunit. Single-step, reagentless detection of the S1 protein is achieved through a binding-induced, concentration-dependent folding of the DNA aptamer that reduces the efficiency of the electron transfer process between the redox tag and the electrode surface and causes a suppression of the resulting amperometric signal. This aptasensor is specific for the target S1 protein with a dissociation constant (*K*_D_) value of 43 ± 4 nM and a limit of detection of 7 nM. We demonstrate that the target S1 protein can be detected both in a buffer solution and in an artificial viral transport medium widely used for the collection of nasopharyngeal swabs, and that no cross-reactivity is observed in the presence of different, non-target viral proteins. We expect that this SWCNT-SPE-based format of electrochemical aptasensor will prove useful for the detection of other protein targets for which nucleic acid aptamer ligands are made available.

## 1 Introduction

The development of rapid, easy-to-use, portable devices for the detection and quantification of specific proteins is key to more efficient and effective laboratory practices and clinical decisions.

Particularly with the emerging risks to public health posed by virus outbreaks and spreading of pathogens, new approaches for the detection of protein antigens are needed that can combine high specificity and sensitivity with a cost-effective and time-saving procedure. Electrochemical biosensors are especially amenable for this purpose because they can be easily engineered into point-of-care (POC) diagnostic devices enabling the rapid detection and quantification of selected targets.^1−5^ The current COVID-19 pandemic caused by the spread of the severe acute respiratory syndrome coronavirus 2 (SARS-CoV-2) has emphasized the importance of having at hand efficient and reliable analytical platforms. This disease continues to claim victims and to determine disruptions in healthcare systems, economies, and social life worldwide.^6−8^ The deployment at a fast pace of different COVID-19 vaccines has had a huge impact on the pandemic by offering protection from severe and acute forms of the disease and therefore helping reduce hospitalization and mortality.^9,10^ Nevertheless, vaccines alone are not able to contain the spread of the virus, and complementary measures must be enforced.^11,12^ Efficient and focused testing is necessary for timely spotting SARS-CoV-2 infection, monitoring the diffusion of the disease, and curbing transmission of the virus.^13,14^ COVID-19 diagnostics has taken center stage in everyday life, especially where proof of a negative test is a requirement for traveling and for accessing public and private spaces.^15,16^ Currently, polymerase chain reaction (PCR) is the gold standard method to detect SARS-CoV-2 infection, enabling quantification of viral RNA with high sensitivity and specificity. However, PCR is reagent-intensive and requires trained personnel and relatively expensive instrumentation. This leads, on the one hand, to waiting times that are not compatible with the highly frequent testing enforced in wealthy countries and determines, on the other hand, a series of practical obstacles to a widespread application in low-resource settings.^17,18^ Cost-effective, time-saving point-of-care (POC) tests that can complement PCR-based methods are therefore much needed. Lateral flow immunoassays enable the rapid detection of a SARS-CoV-2 antigen by using low-cost, portable hardware.^19^ However, their relatively low sensitivity and specificity is a limit to their ability to provide unambiguous information for accurate diagnostics and therefore to their potential to guide healthcare and policy measures.^20^ In response to these limitations, several electrochemical COVID-19 immunosensors were recently developed that enabled ultrasensitive detection of SARS-CoV-2 antigen proteins.^21−24^ A particular format of electrochemical sensors is E-DNAs. These are rapid, simple, reagent-free sensors that leverage target-induced conformational or structural changes in a DNA-based architecture or in a DNA aptamer to generate a measurable output following variation of the electron transfer efficiency between a redox reporter and the electrode surface.^25−27^ In the context of COVID-19, Kelley and coworkers achieved detection of viral particles within 5 min through a chronoamperometry strategy based on electrodes modified with hybrid DNA-antibody receptors targeting the SARS-CoV-2 spike (S) protein displayed on the virion surface.^28^ In aptamer-based E-DNAs, an electrochemical signal is generated when a binding-induced change in the structure folding of a redox-tagged aptamer leads to a change in the relative position of the redox reporter with respect to the electrode surface.^29^ Recently, Idili et al. applied this strategy to COVID-19 diagnosis and performed electrochemical detection of the SARS-CoV-2 S protein by using gold electrodes modified with a DNA aptamer engineered to undergo a binding-induced conformational change.^30^ In such a context, here we report the development on an electrochemical sensing platform based on cheap, commercially available single-walled carbon nanotube screen-printed electrodes (SWCNT-SPEs) functionalized with a redox-tagged DNA aptamer selected against the receptor binding domain (RBD) of the SARS-CoV-2 spike protein S1 subunit.^31^ Binding-induced folding of this DNA aptamer in the presence of the target S1 protein leads to a concentration-dependent suppression in the registered amperometric signal. We demonstrate that this aptasensor specifically recognizes and detects the target S1 protein both in a buffer solution and in an artificial complex matrix, requiring only a few hours of incubation and no additional reagents.

## 2 Experimental Section

### 2.1 Materials

Sodium chloride (NaCl; ACS reagent ≥99.0%), sodium bicarbonate (NaHCO_3_; ACS reagent ≥99.7%), disodium hydrogen phosphate (Na_2_HPO_4_; anhydrous 99.99% Suprapur), potassium dihydrogen phosphate (KH_2_PO_4_; 99.995% anhydrous basis, Suprapur), sodium dodecyl sulfate (SDS; ACS reagent ≥99.0%), Trizma Base (puriss. p.a., ≥99.7%), *N*-(3-dimethylaminopropyl)-*N′*-ethylcarbodiimide hydrochloride (EDC; purum, ≥98.0% (AT)), *N*-hydroxysuccinimide (NHS; purum, ≥97.0% (AT)), 4-morpholineethanesulfonic acid monohydrate (MES; BioXtra, ≥99.0% (T)), pyrene (98%), ethanolamine (ACS reagent ≥99.0%), hydrochloric acid (HCl; 37% w/v), potassium chloride (KCl; ACS reagent, ≥99.0%), sodium hydroxide (NaOH; ACS reagent, ≥97.0%), dimethyl sulfoxide (DMSO; anhydrous ≥99.9%) were purchased from Sigma-Aldrich (Milan, Italy). Viral Transport Medium (VTM) was purchased from CleaniSciences (Guidonia Montecelio, Italy). The following synthetic probes were purchased from Metabion International AG (Plannegg, Germany): redox-tagged SARS-CoV-2 aptamer: 5′-AttoMB2-CGC AGC ACC CAA GAA CAA GGA CTG CTT AGG ATT GCG ATA GGT TCG GTT TTT – C7 Amino-3′; SARS-CoV-2 aptamer: 5′-CGC AGC ACC CAA GAA CAA GGA CTG CTT AGG ATT GCG ATA GGT TCG GTT TTT-C7 Amino-3′; redox-tagged thrombin aptamer: 5′-AttoMB2-TAA GTT CAT CTC CCC GGT TGG TGT GGT TGG T-C7 Amino-3′; redox-tagged PDGF aptamer: 5′-AttoMB2-CAG GCT ACG GCA CGT AGA GCA TCA CCA TGA TCC TG-C7 Amino-3′. SARS-CoV-2 Spike protein (S1) and Influenza A H1N1 (A/California/04/2009 (H1N1)) were purchased from Twin Helix Srl (Milan, Italy). Recombinant Coronavirus Spike Protein MERS-CoV S1 was purchased from Vinci-Biochem Srl (Vinci, Italy). Buffers were prepared as follows: MES buffer: 0.1 M MES (pH adjusted to 5 with NaOH); Tris-buffered saline (TBS): 0.1 M Trizma base (pH adjusted to 7.4 with HCl); carbonate buffer (CB): 0.1 M NaHCO_3_, 0.1% w/v SDS (pH adjusted to 9 with NaOH); phosphate-buffer saline (PBS): 1.37 M NaCl, 0.08 M Na_2_HPO_4_, 0.027 M KCl, 0.012 M KH_2_PO_4_ (pH adjusted to 7.4 with HCl); reading buffer (RB): PBS. Single-walled carbon nanotube screen-printed electrodes (SWCNT-SPEs) were purchased from Metrohm Italiana Srl (Varese, Italy).

### 2.2 Aptamer Immobilization on SWCNT-SPEs

The SWCNT surface was initially treated with 0.2 M EDC and 0.05 M NHS in MES buffer (50 μL) for 30 min to activate the carboxylic groups of the carbon nanotubes, followed by rinsing with water. Subsequently, 50 μL of redox-tagged SARS-CoV-2 aptamer (500 nM) solution in CB was deposited onto the SWCNT electrode and left incubating for 2 h. The electrode was then thoroughly washed with water. A capping step of 30 min using ethanolamine in TBS (50 mM) was carried out to quench the unreacted active ester groups. Next, the surface was washed with TBS. A solution of pyrene as a backfilling agent in DMSO (500 nM) was deposited on the electrode (50 μL) for 30 min, after which the electrode was rinsed first with DMSO and then with water.

### 2.3 Determination of the Aptamer Surface Density

Fluorescence spectroscopy was used to estimate the surface density of the covalently immobilized SARS-CoV-2 aptamer. The emission of the redox-tag AttoMB2 conjugated to the aptamer sequence was collected in solution at λ_Em_ = 680 nm (λ_Ex_ = 650 nm) before (*I_i_*) and after (*I_f_*) the aptamer immobilization on the electrode (Figure S2). The Δ*I_i-f_* percentage was used to estimate the number of aptamer probes per mm^2^ tethered to the CNTs (see eqs 1–6 in the SI). The measurements were replicated three times, and the value obtained is reported as the mean value ± standard deviation.

### 2.4 Detection of S1 Protein

A solution of S1 protein at different concentrations (0.3, 1, 3, 10, 25, 30, 50, 100, 300, and 500 nM) in PBS was incubated for 2 h, at room temperature, on the electrode surface, followed by rinsing with PBS. Next, 50 μL of PBS was deposited onto the surface and the electrochemical measurement was performed by acquiring a DPV scan with the following parameters: potential range from −1.1 to −0.2 V; step potential +0.00495 V; modulation amplitude +0.04995 V; modulation time 0.102 s; interval time 0.4 s. The same protocol was applied during specificity studies, when a thrombin aptamer and a PDGF aptamer, respectively, were immobilized onto the electrode surface and exposed to S1 protein, as well as when MERS-CoV S1 and Influenza-A H1N1 proteins were incubated onto the electrode surface previously functionalized with the SARS-CoV-2 aptamer. This protocol and acquisition parameters were also used for measurements of S1 protein diluted in VTM as the binding buffer (50 and 100 nM).

### 2.5 Competitive Experiments of S1 Protein Binding

The electrode surface was functionalized as described above with a redox-tagged SARS-CoV-2 aptamer. The target S1 protein (100 nM concentration, PBS) was incubated with an unlabeled version of the same SARS-CoV-2 aptamer sequence for 2 h at room temperature. This solution was then transferred onto the electrode surface and left incubating for 2 h. Then, the electrode surface was washed with PBS and 50 μL of PBS were deposited onto the surface to carry out the electrochemical measurement by acquiring a DPV scan with the same parameters reported in Section 2.3.

### 2.6 Data Analysis

The current signals obtained from the electrochemical measurements as a function of the corresponding S1 protein concentrations were analyzed in OriginPro (OriginLab) by using the Langmuir-type equation reported below:
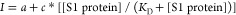
where *K*_D_ is the dissociation constant and *a* and *c* are the fitting parameters of the Langmuir equation. All the measurements were replicated three times, and all the figures show the mean values ± standard deviations. The limit of detection (LOD) and limit of quantification (LOQ) were determined according to Eurachem guidelines (https://www.eurachem.org).

## 3 Results and Discussion

### 3.1 Immobilization of SARS-CoV-2 DNA Aptamer

**Figure 1:**
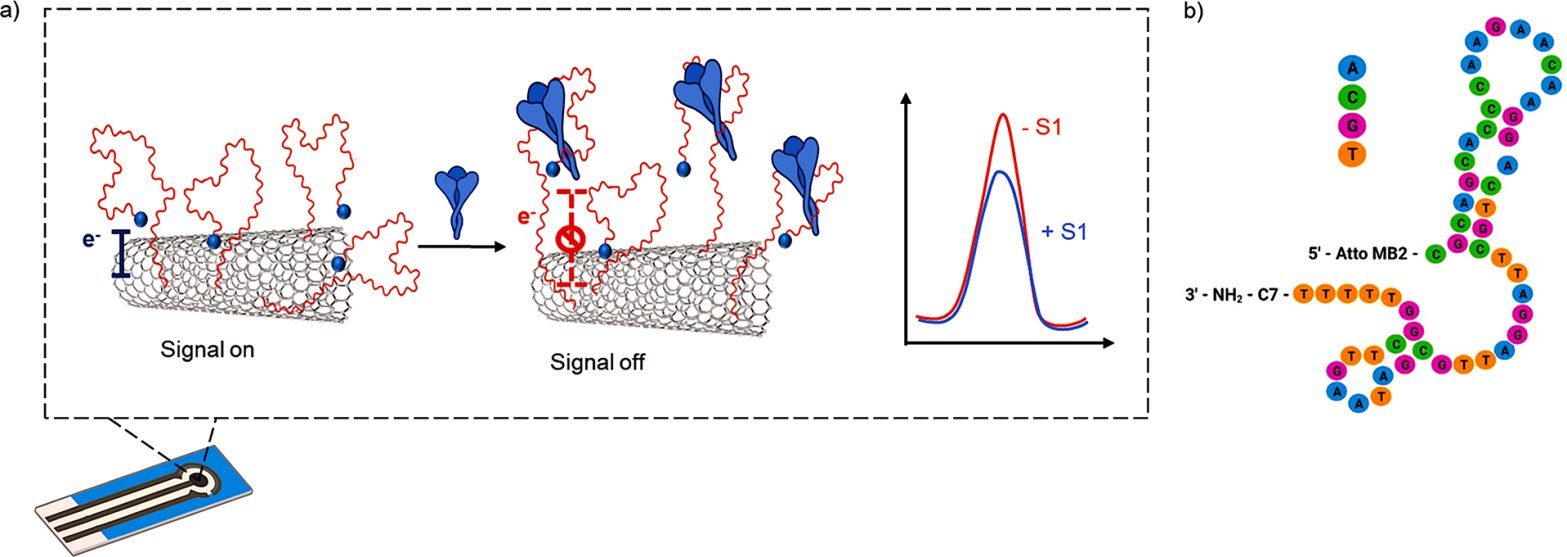
(a) Schematic illustration of the working principle of the aptasensor based on the conformational change in a redox-tagged SARS-CoV-2 aptamer upon interaction with the target S1 protein. A suppression of the output current is observed in the presence of S1 protein because of the structural rearrangement in the aptamer folding that moves the redox reporter away from the electrode surface. (b) Secondary structure of the AttoMB2-modified SARS-CoV-2 aptamer used in this work, based on a recently discovered aptamer sequence.^31^

Several aptamers have been recently artificially evolved that can specifically bind to the S1 protein of SARS-CoV-2 and can potentially support new therapeutic strategies for COVID-19.^31−34^ DNA aptamers can be used in lieu of antibodies as specific recognition elements in the development of SARS-CoV-2 biosensors, enabling a range of sensing platforms with optical or electrochemical readout.^30,32,35−40^ Besides their use as “static” synthetic receptors, aptamers are useful as “dynamic” probes in folding-based E-DNAs. In this work, we propose a novel E-DNA format for the single-step, reagent-free detection of the S1 protein that leverages cheap, commercially available SWCNT-SPEs as the sensing substrate. SWCNT-SPEs are a promising electrochemical platform because they are cheaper than standard gold electrodes, they show intrinsic electrocatalytic properties and a high conductivity that both improve electron transfer processes at the interface and provide enhanced amplification of current signal, and they offer a larger surface area for immobilization of an increased number of probes and receptors.^41−43^ The sensing mechanism of our sensor is based on a rearrangement in the redox-tagged aptamer structure induced by binding to the target S1 protein, which translates in a measurable electrochemical output. A fast electron transfer is observed in the absence of the target protein because of the π–π interactions between the redox-tagged DNA aptamer and the carbon nanotubes that bring the AttoMB2 tag in close proximity to the electrode surface. Conversely, in the presence of the target, the aptamer undergoes a binding-induced structural folding that increases the distance between the redox tag and electrode surface, thus reducing the efficiency of the electron transfer process and causing a decrease in the registered amperometric current (Figure 1).

To fabricate the aptasensor, we used a modified version of a recently published SARS-CoV-2 DNA aptamer that specifically binds to the receptor binding domain (RBD) of the SARS-CoV-2 spike protein S1 subunit.^31^ We conjugated the aptamer at its 3′ terminus with an AttoMB2 tag, a derivative of the common redox tag methylene blue that generates an electrochemical signal measured by DPV and also allows for performing fluorescence spectroscopy measurements based on its emission properties. A free amine group was introduced instead at the 5′ terminus of the aptamer sequence to allow covalent anchoring to the electrode surface through the formation of an amide bond with the carboxylic groups present on the SWCNTs, using EDC/NHS as a coupling mixture. This coupling reaction had been already used in several works^43−45^ and was further optimized by diluting the aptamer in carbonate buffer (pH = 9) with 0.1% SDS to increase the wettability of the CNT hydrophobic surface. The concentration of the aptamer in the carbonate buffer solution used for covalent immobilization on the electrode surface was 500 nM, based on a previous work in which we investigated different concentrations of biotinylated amine-modified DNA probes to maximize surface functionalization, using an enzyme-based reaction to generate an electrochemical signal proportional to the amount of DNA probes attached to the electrode surface (Figure S1).^44^ The surface density of the aptamer was estimated by means of fluorescence spectroscopy following the emission of the AttoMB2 tag at λ = 680 nm before and after the immobilization of the aptamer onto the surface (Figure S2). A value of (1.7 ± 0.4) · 10^13^ aptamer molecules per mm^2^ was obtained, which is higher than the average values generally found in the literature for E-DNAs based on gold electrode substrates.^46,47^ The electrode surface was eventually treated with a solution of pyrene in DMSO as a backfilling agent to minimize non-specific adsorption of the biomolecules contained in the analyzed samples. This pyrene-based backfilling strategy was developed and applied in previous works and proved particularly efficient in reducing non-specific adsorption of non-target biological molecules when compared to other common blocking strategies such as BSA.^43,44^

### 3.2 S1 Protein-Aptamer Interaction Analysis

**Figure 2:**
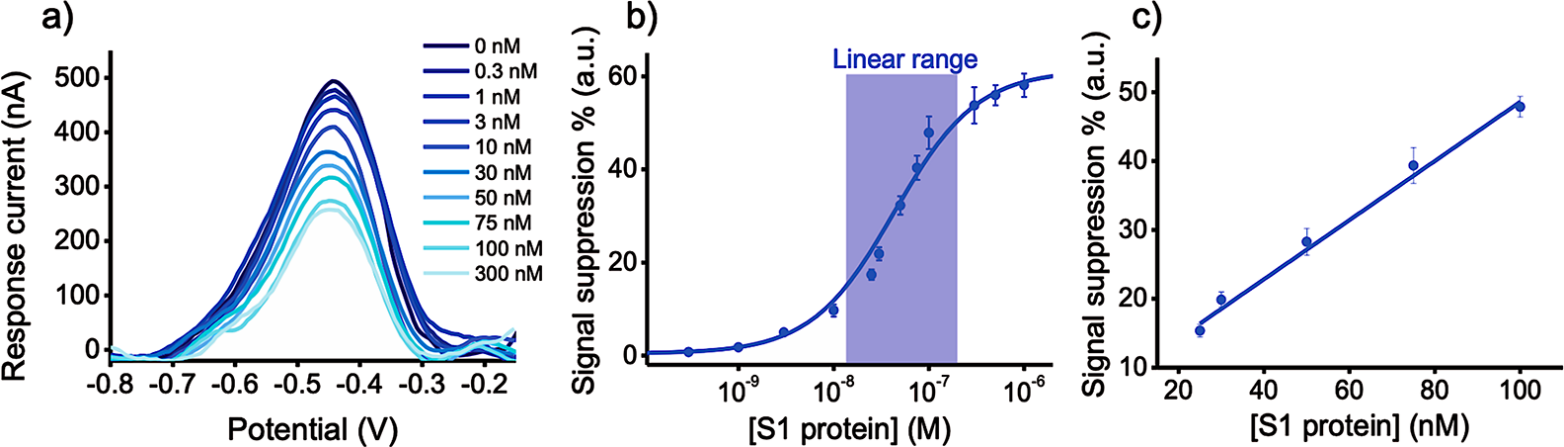
(a) DPV voltammograms obtained in the presence of S1 SARS-CoV-2 protein in the concentration range 0.3–300 nM. (b) Binding curve based on a Langmuir-type equation describing the response current as a function of the S1 protein concentration. Highlighted is the concentration range in which response is linear. (c) Calibration curve obtained by linear fit of the response current values in the 20–100 nM S1 protein concentration range (in all the figures, data are reported as mean value ± SD, *n* = 3).

The sensor was then exposed to increasing concentrations of S1 protein from 0.3 to 500 nM in PBS buffer, and DPV voltammograms were acquired. We observed a signal-off behavior, i.e., a decrease in the amperometric signal given by the AttoMB2 redox reporter, after interaction of the aptamer with the different concentrations of the S1 protein, which likely depends on AttoMB2 moving away from the electrode surface upon target-induced folding of the DNA aptamer (Figure 2a). The decrease in the registered amperometric current can be expressed as signal suppression % with respect to the current signal measured in the absence of S1 protein. The obtained data were analyzed by means of a Langmuir-type binding curve model and the dissociation constant value between the aptamer attached to the electrode surface and its target S1 protein was *K*_D_ = 43 ± 4 nM (Figure 2b). This is in good agreement with the affinity measured in solution by means of fluorescence spectroscopy reported for the original aptamer (*K*_D_ = 45 ± 10 nM).^31^ In particular, it was possible to obtain a limit of detection (LOD) of 7 nM and a limit of quantification (LOQ) of 21 nM (Figure 2c).

### 3.3 Specificity in the S1 Protein–Aptamer Interaction

**Figure 3:**
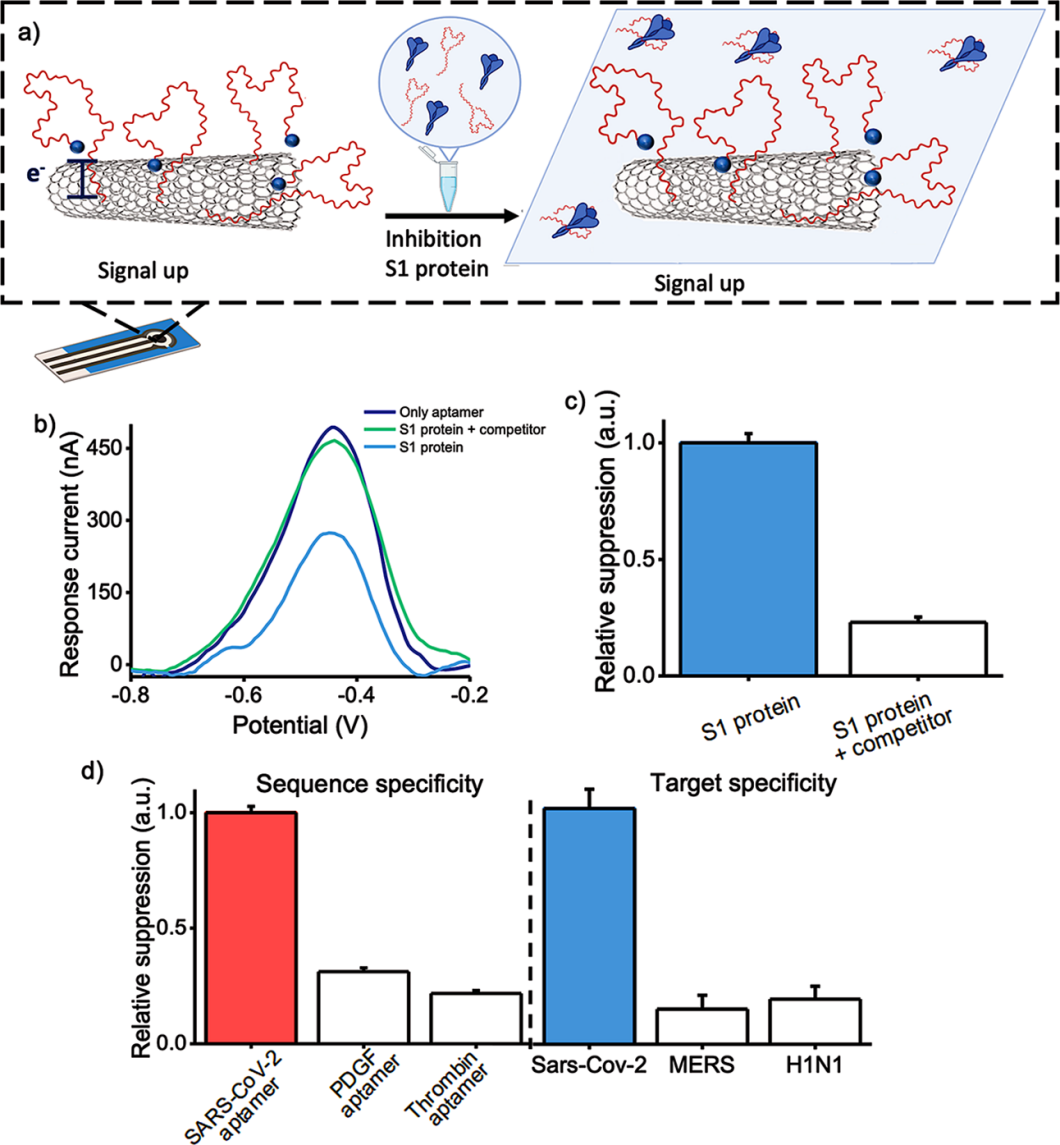
(a) Schematic illustration of the aptasensor behavior when pre-treating the target S1 protein with the same SARS-CoV-2 aptamer, lacking the redox reporter, as a competitor in solution. (b) DPV voltammograms in the absence of the target protein (only aptamer, dark blue line), in the presence of 100 nM S1 protein pre-incubated with an excess of competitor aptamer (+ S1 protein + competitor, green line), and in the presence of the S1 protein at 100 nM concentration (+ S1 protein, light blue line). (c) Relative signal suppression obtained when measuring the amperometric current in the presence of S1 protein 100 nM (light blue bar) and in the presence of the same protein incubated with the aptamer competitor (white bar). (d) Specificity of the sensor evaluated by using different aptamer sequences immobilized onto the electrode surface (SARS-CoV-2, PDGF, and thrombin aptamer) in the presence of S1 protein at 100 nM concentration in PBS (left panel), and by using different proteins at 100 nM concentration in PBS (S1 SARS-CoV-2, S1 MERS and H1N1) when the SARS-CoV-2 aptamer is immobilized onto the electrode surface (right panel) (in all the figures data are reported as mean value ± SD, *n* = 3).

Further evidence of the interaction between the DNA aptamer and the target S1 protein, which supports the switching mechanism proposed for the developed aptasensor, was achieved by means of a competitive experiment^48^ in which the S1 protein (100 nM) was pre-incubated in solution with a non-redox-tagged version of the same SARS-CoV-2 aptamer sequence, utilized as a competitor. Binding of this aptamer to the RBD of the target S1 protein would result in impeding subsequent binding to the redox-tagged aptamer on the electrode surface (Figure 3a). When the S1 protein was pre-treated with the aptamer competitor, only a slight change in the current signal was observed compared with that in the absence of the protein (Figure 3b), and the signal suppression was only a fraction (∼17%) of that obtained in the absence of the competitor at the same concentration of S1 protein (Figure 3c). This suggests that the RBD of the S1 protein was already occupied by the competitor inhibiting further binding, and the aptamer immobilized on the surface retained its conformational structure maintaining the redox reporter AttoMB2 close to the electrode surface.

The same experiment conducted extending the incubation time up to 24 h led to no significant effects in the measured current signal (*p* > 0.05) and therefore in the calculated relative signal suppression % (Figure S3). This indicated that an incubation time of 2 h was enough for achieving tight binding of the aptamer to its target spike protein, which indirectly suggested that the same incubation time would work also when the binding event involves the redox-tagged aptamer immobilized on the electrode surface.

We then tested the ability of our aptasensor to recognize its target S1 protein in a specific manner. To do so, we first exposed it to two different viral proteins from Middle East respiratory syndrome coronavirus (MERS-CoV) and Influenza A H1N1 as model potential interfering pathogens. At saturating concentrations (100 nM), signal suppression of only ∼15% when using the MERS-CoV protein and of ∼19% when using the Influenza A H1N1 protein, respectively, were registered with respect to the signal suppression % obtained with the target S1 protein (Figure 3d, right). To have further confirmation that the changes in the current signal were specific to binding of the S1 protein to its cognate aptamer ligand, we immobilized two different aptamer sequences, a thrombin DNA aptamer and a prostate-derived growth factor (PDGF) DNA aptamer, on the electrode surface, and exposed them to the S1 protein. We selected these aptamers because they had been previously used in the development of folding-based electrochemical sensors.^49,50^ In the presence of 100 nM S1 protein, a 15% signal suppression with the thrombin aptamer and a 21% signal suppression with the PDGF aptamer, with respect to the signal suppression obtained using the correct S1 protein–aptamer couple, were observed, showing that cross-reactivity was minimal (Figure 3d, left).

### 3.4 S1 Protein Detection in Viral Transport Medium

To test the aptasensor ability to function in a more challenging matrix mimicking a real-world scenario, we investigated its performance when using S1 protein samples in viral transport medium (VTM), an artificial complex matrix used in the clinic for rapid antigen detection tests. Its composition includes physiologically balanced isotonic buffered solution at neutral pH, a stabilizing protein component, and antibacterial and antifungal agents.

**Figure 4:**
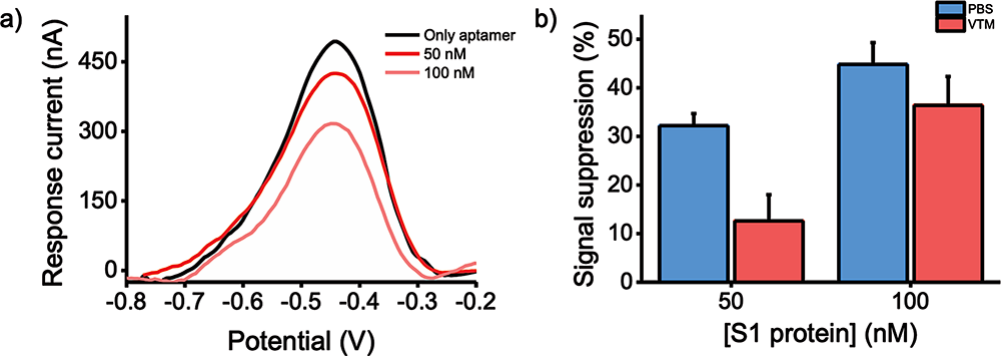
(a) DPV voltammogram obtained in the presence of target S1 protein in VTM at 50 and 100 nM. (b) Histograms showing the sensor performance, expressed as signal suppression %, when the S1 protein is dissolved in PBS (blue bars) or VTM (red bars) (mean value ± SD, *n* = 3).

When the aptasensor was exposed to VTM-based solutions of S1 protein at concentrations of 50 and of 100 nM, a decrease in the measured current signal was observed (Figure 4a). Values of signal suppression % of 15% and 36% were obtained (Figure 4b). These values indicate that the aptasensor is still capable of recognizing and detecting the target S1 protein in a concentration-dependent manner, although the use of a complex matrix such as VTM has an impact on its analytical performances and leads to signal suppression values that are lower than those obtained using S1 protein samples in PBS (Figure 4b). Among the many possible effects, a change in the ionic strength of the medium due to the high salt concentration in the VTM can be a major cause of interference as this is known to affect the binding affinity properties of DNA aptamers.^51,52^ However, we note that the response of the aptasensor was only slightly affected by the use of undiluted VTM, especially when compared with analogous effects observed with other COVID-19 biosensors.^53^ For a better contextualization of the performances of the developed aptasensor, we have reported in Table 1 the characteristics of several recent biosensors with relevance to our sensor format, that is, consisting of a nanostructured substrate and targeting a protein antigen from SARS-CoV-2.

**Table 1:** Characteristics of Recently Developed Nanostructured Biosensors for SARS-CoV-2 Detection

nanostructured substrate	transduction	recognition element	SARS-CoV-2 target element	LOD	reference
carbon nanotubes	optical	ACE2 receptor	RBD	9.5 nM	(54)
chitosan/graphitic carbon	photo-electrochemical	aptamer	RBD	0.12 nM	(55)
nitride cadmium selenide					
gold-coated platinum nanoparticles	electrochemical	aptamer	nucleocapsid protein	8.33 pg/mL	(56)
carbon nanotube FET	electronic	SARS-CoV-2 S1 antibody	spike protein	4.12 fg/mL	(57)
single-walled carbon nanotubes	electrochemical	aptamer	RBD	7 nM	this work

## 4 Conclusions

Folding-based E-DNAs that use target-induced changes in an aptamer structure have been previously designed and enabled protein detection on gold electrodes.^30,58,59^ In this context, we investigated a novel folding-based electrochemical biosensor based on cheap, highly conductive SWCNT-SPEs as a new substrate for aptamer-based E-DNAs, allowing the development of a rapid and reagent-free electrochemical sensing platform for the single-step detection of the SARS-CoV-2 S1 protein. This biosensor benefits from the advantageous physicochemical properties of SWCNT-SPEs and leverages a folding-based mechanism that results in significant changes in the measurable amperometric current upon specific binding of the S1 protein to a DNA aptamer ligand. The obtained LOD and LOQ in the low nanomolar range, together with the high specificity for the target protein and the low cross-reactivity in the presence of interfering viral proteins, all suggest the potential use of this aptasensor as a compact, easy-to-use sensing device for the detection of the S1 protein in a buffer solution or in a complex biological matrix. As the expected concentration of S1 spike protein in an aqueous or VTM-based sample can largely vary depending on sample pre-treatment, which can include lysis, pre-concentration, or purification processes, the “fitness for purpose” of the aptasensor to analyze an unknown sample should be evaluated within a given context and specific working conditions.^60^ The aptamer sequence used in this work was demonstrated to fold up and bind to the Spike protein on the virion surface, inducing neutralization of the whole SARS-CoV-2 virus and blocking cell infection *in vitro.*(31) Based on these results, it is likely that the same aptamer sequence used in the development of our aptasensor would be able to interact and bind to the spike protein displayed on the surface of the virion, therefore enabling detection of the whole SARS-CoV-2 virus. Further studies will be needed to perform validation of the sensor using spiked and/or real samples in VTM to determine analytical parameters, including an LOD and an LOQ, relevant to the whole virus as the target analyte. We conclude that the versatility and the simplicity of our design, in which a DNA aptamer as a specific, dynamic recognition element is combined with a CNT-based electrode substrate, could inspire the development of many more electrochemical platforms of this kind. As new aptamers designed to bind to desired target biomolecules are generated through SELEX, a variety of new sensors can be envisioned with applications in a wide range of fields.

## Supporting Information Available

Additional details on electrode surface functionalization and incubation protocols (PDF)
