# Supplementary material for: A Folding-Based Electrochemical Aptasensor for the Single-Step Detection of the SARS-CoV-2 Spike Protein
Source: ACS Appl Mater Interfaces. 2022 Apr 21;14(17):19204–11. doi: 10.1021/acsami.2c02405 (PMC9045037; doi:10.1021/acsami.2c02405)
Supplement: Supplementary file 1 — am2c02405_si_001.pdf [file am2c02405_si_001.pdf]

SUPPORTING INFORMATION

A Folding-Based Electrochemical Aptasensor for the Single-Step Detection of the SARS-CoV-2 Spike Protein

Federica Curti,[a,b] Simone Fortunati,[a] Wolfgang Knoll,[b,c] Marco Giannetto,[a] Roberto Corradini,[a] Alessandro Bertucci,[a*] and Maria Careri[a]

[a] Department of Chemistry, Life Sciences and Environmental Sustainability, University of Parma, 43124 Parma, Italy.

[b] Biosensor Technologies, AIT-Austrian Institute of Technology GmbH, Konrad-Lorenz-Straße 24, 3430 Tulln an der Donau, Austria

[c] Department of Scientific Coordination and Management, Danube Private University, A-3500 Krems, Austria

* Corresponding Author Email:

alessandro.bertucci@unipr.it

Table of contents

1. Supporting Figures

1. Supporting Figures

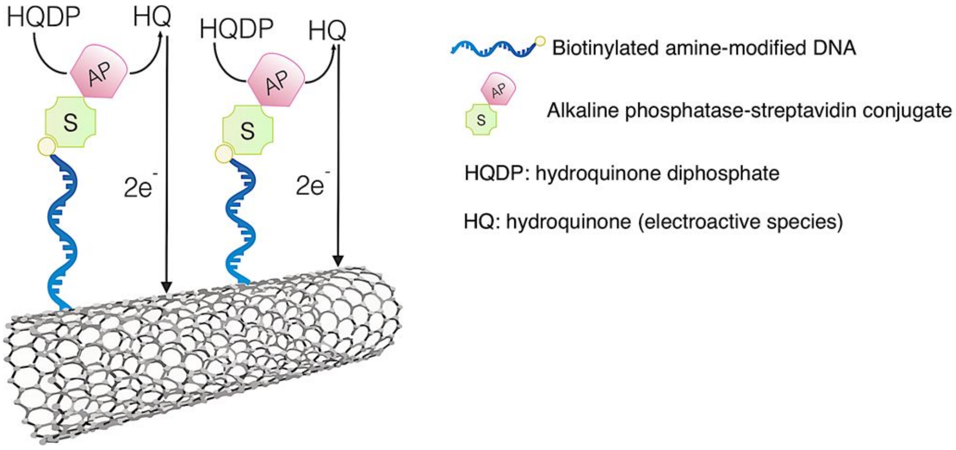

Figure S1. Schematic drawing of the generation of an enzyme-based amplified current signal using biotinylated DNA probes complexed with alkaline phosphatase-streptavidin conjugates.[1]

[1] Fortunati, S.; Vasini, I.; Giannetto, M.; Mattarozzi, M.; Porchetta, A.; Bertucci, A.; Careri, M.; Controlling Dynamic DNA Reactions at the Surface of Single-Walled Carbon Nanotube Electrodes to Design Hybridization Platforms with a Specific Amperometric Readout. *Anal. Chem.* **2022,** *94*(12), 5075-5083.

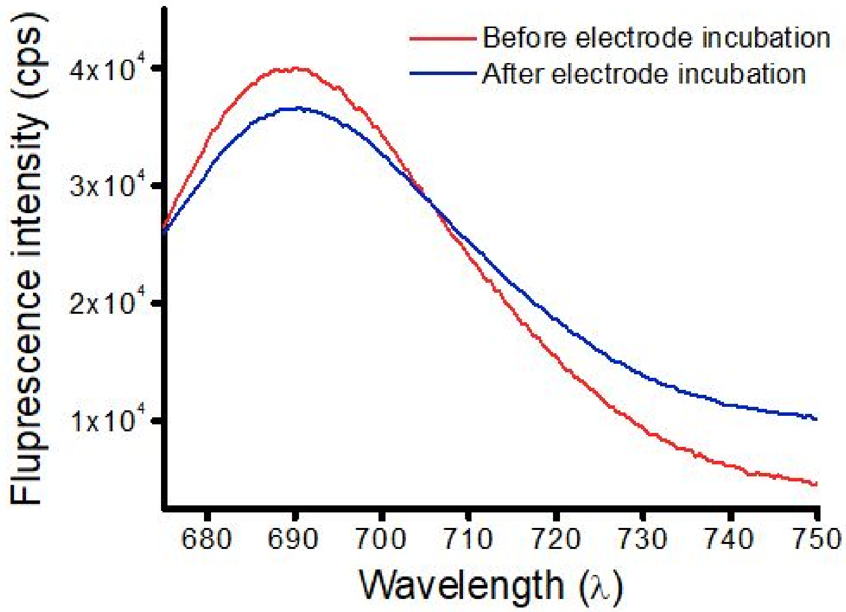

Figure S2. Fluorescence emission spectra of a solution of AttoMB2-labelled amine-modified aptamer in carbonate buffer (500 nM) before (red line) and after (blue line) incubation on an NHS-functionalized SWCNT-SPE (λex = 670 nm).

(1) $\Delta CPS = 38637 — 35294 = 3343 \Rightarrow \frac{3343}{38637} \times 100\% = 8.7\%$

(2) $moles\ AttoMB2\text{-}aptamer = (500 \times 10^{-9}\ M) \times (100 \times 10^{-6}\ L) = 5 \times 10^{-11}\ mol$

(3) $DNA\ moles\ attached\ to\ the\ surface = (5 \times 10^{-11}\ mol) \times 8.7\% = = 4.4 \times 10^{-12}\ mol$

(4) $Electrode\ surface\ area = \pi(4\ mm)^{2} = 12{,}6\ mm^2$

(5) $moles\ electrode\ surface = (4.4 \times 10^{-12}\ mol)\ (12{,}6\ mm^2\ ) = 1.7 \times 10^{-13}\ mol\ mm^2$

(6) $DNA\ molecules\ electrode\ surface = (1.2 \times 10^{-12}\ mol) \times NA\ (12{,}6\ mm^2) = 1.7 \times 10^{13} molecules\ mm^2$

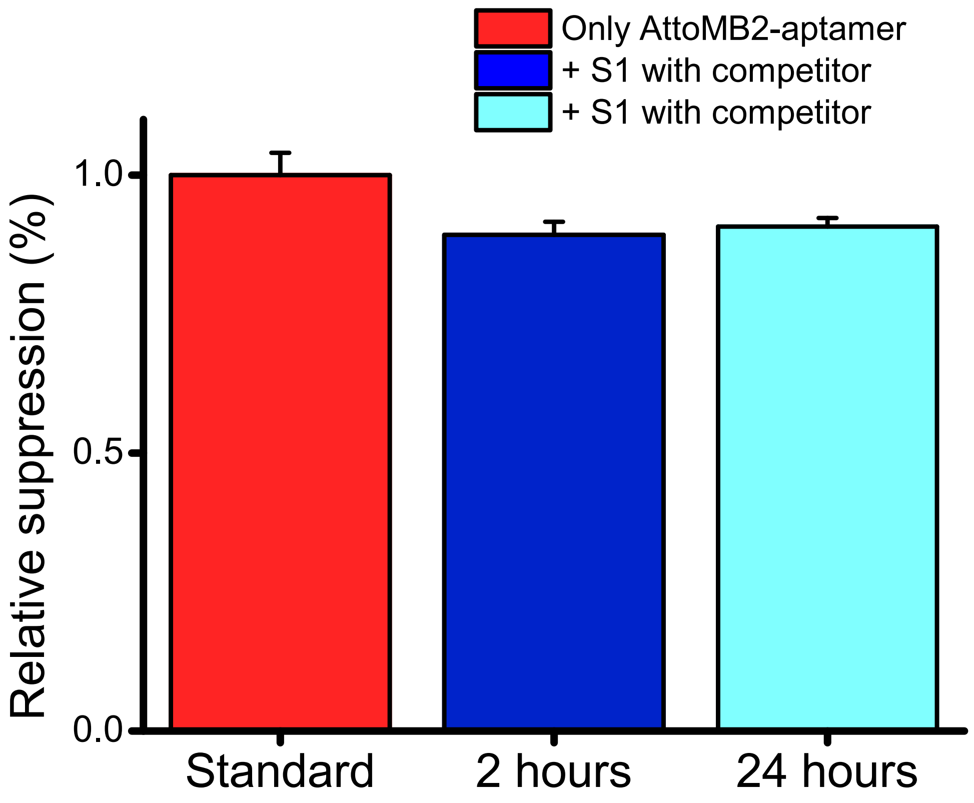

Figure S3. Relative suppression obtained when measuring the amperometric current in the absence of S1 protein (red bar) and in the presence of the same protein (100 nM) incubated with an unlabeled aptamer as an inhibitor (500 nM) for 2 hours (blue bar) and for 24 hours (light blue bar). The difference in relative suppression % at 2 h and 24 h is non-significant (mean ± SD, n =3, $p > 0.05$).
